# Electricity Usage Scheduling in Smart Building Environments Using Smart Devices

**DOI:** 10.1155/2013/468097

**Published:** 2013-12-26

**Authors:** Eunji Lee, Hyokyung Bahn

**Affiliations:** ^1^Department of EECS, University of Michigan, Ann Arbor, MI 48105, USA; ^2^Department of Computer Engineering, Global Top 5 Research Institute, Ewha University, Seoul 120-750, Republic of Korea

## Abstract

With the recent advances in smart grid technologies as well as the increasing dissemination of smart meters, the electricity usage of every moment can be detected in modern smart building environments. Thus, the utility company adopts different price of electricity at each time slot considering the peak time. This paper presents a new electricity usage scheduling algorithm for smart buildings that adopts real-time pricing of electricity. The proposed algorithm detects the change of electricity prices by making use of a smart device and changes the power mode of each electric device dynamically. Specifically, we formulate the electricity usage scheduling problem as a real-time task scheduling problem and show that it is a complex search problem that has an exponential time complexity. An efficient heuristic based on genetic algorithms is performed on a smart device to cut down the huge searching space and find a reasonable schedule within a feasible time budget. Experimental results with various building conditions show that the proposed algorithm reduces the electricity charge of a smart building by 25.6% on average and up to 33.4%.

## 1. Introduction

Modern power systems are evolving to a highly interconnected infrastructure called smart grid, which is an emerging technology that combines the traditional electricity supply infrastructure with information technologies. Due to the recent advances in smart grid as well as the increasing dissemination of smart meters, the electricity usage of every moment in a building can be detected and then transferred to the utility company [1, 2]. Thus, the utility company can adopt different price of electricity at each time slot of a day. The prices are usually higher during peak hours such as the afternoon on hot days in the summer [3]. The rationale behind this is to shift the electricity demand away from the time of peak load, expecting a consumer-side reaction according to the change of prices.

In these situations, consumers can detect the price change and then reduce the electricity charges by controlling their own loads [4–7]. However, it is generally inconvenient and impractical for consumers to manually keep track of constantly changing prices and take actions accordingly [3]. To achieve the full economic benefit of dynamic pricing, it is imperative that electric devices be equipped with an automatic price-aware scheduling mechanism that requires minimal action from the consumers.

In this regard, smart sockets have been developed [8]. A smart socket is placed between an appliance and an electricity outlet and monitors the electricity usage information of the appliance; it also has an ability to communicate with smart meters and controls the electricity state of the appliance. More recently, as the standard communication protocols for smart appliances over the home-area network (HAN) are established, smart appliances themselves are being developed to have an ability to communicate with smart meters or control systems to be managed remotely [9–12].

**Figure 1:**
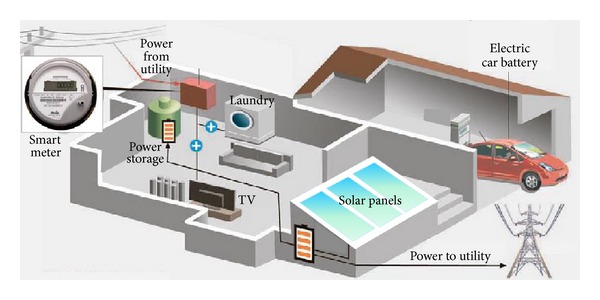
An electricity usage scheduling architecture with a smart meter.

Accordingly, demand-side management can be performed more precisely [13]. For example, various power consuming devices in a smart building, such as smart appliances, HVAC (heating, ventilating, and air conditioning) systems, LED illumination, electric car batteries, and fuel cells, can be scheduled dynamically according to the time-varying electricity prices. When the price is low, all electricity demands are in their normal mode, while the scheduler can switch some of them to low-power modes when the price becomes high. As smart buildings would be equipped with renewable energy sources such as solar panels or a windmill power generator, there are alternative energy sources that can be provided. The scheduler also needs to consider this alternative source of electricity during scheduling.  Figure 1 describes a basic environment of the electricity usage scheduling with a smart meter in a smart building.

This paper presents a new electricity usage scheduling algorithm for smart buildings that adopts real-time pricing of electricity. Specifically, we formulate the electricity usage scheduling problem as a real-time task scheduling problem and show that it is a complex search problem that has an exponential time complexity. The proposed scheme uses an efficient heuristic based on the evolutionary theory, which cuts down the huge searching space and finds a reasonable schedule within a feasible time budget. Specifically, a collaborative scheduling with genetic algorithms is performed on a smart device to minimize the electricity charges.

Experimental results with various electricity demand conditions show that the proposed algorithm reduces the electricity charges of a smart building by 25.6% on average and up to 33.4%. This can contribute to alleviating the disparity of power usage within a day by reducing the electricity usage at peak time. It will be also helpful to the success of dynamic pricing, which highly depends on the consumer's actual response to the time-varying prices.

The remainder of this paper is organized as follows.  Section 2 describes the motivation of this research.  Section 3, then, presents the proposed scheduling algorithm. Then, Section 4 presents the experimental results to assess the effectiveness of the proposed algorithm.  Section 5 summarizes related works of this research, and Section 6 finally concludes this paper.

## 2. Motivations 

**Figure 2:**
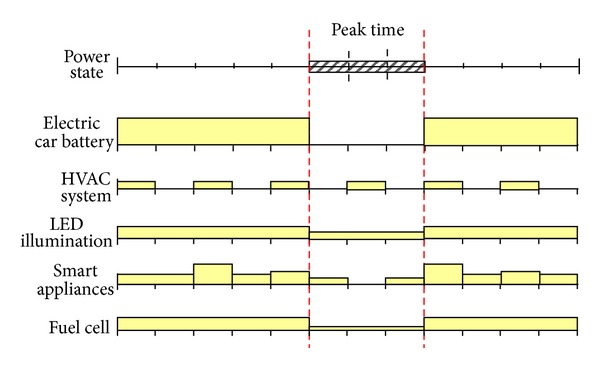
An example of electricity usage scheduling.

Figure 2 shows an example of electricity usage scheduling for each electricity demand as time goes on. In the figure, peak time represents the time period at which the price of electricity becomes high. As shown in the figure, it is desirable to change the state of each device to a low-power mode during the peak time. Meanwhile, each device has its own constraints such as different power modes, minimum activation periods, operation length, deadline, and preemptibility. In reality, the scheduling problem is very complicated as each device should be cooperated to find an optimal schedule. This complex problem space should be searched by optimization techniques such as evolutionary computation methods. The complexity of the problem can be summarized as the following two points.

*Progressive Stage System*. Even at a single time slot, it is not the case that the price of electricity increases in proportion to the usage of power but jumps up dramatically when the usage becomes over a certain threshold value. Thus, the electricity charge becomes high when simultaneous demands from many devices are concentrated on a specific time interval even at an off-peak period. Therefore, a cooperated scheduling of each electric device should be performed not to exceed the progressive threshold. For example, there are three subsections in the peak time of Figure 2 and as the HVAC system is inactivated in the first sub-section, other devices can be a little more activated. In contrast, as the HVAC system is activated in the second sub-section, the scheduling system changes the state of smart appliances to low-power modes not to exceed the progressive threshold of total power usage.

**Figure 3:**
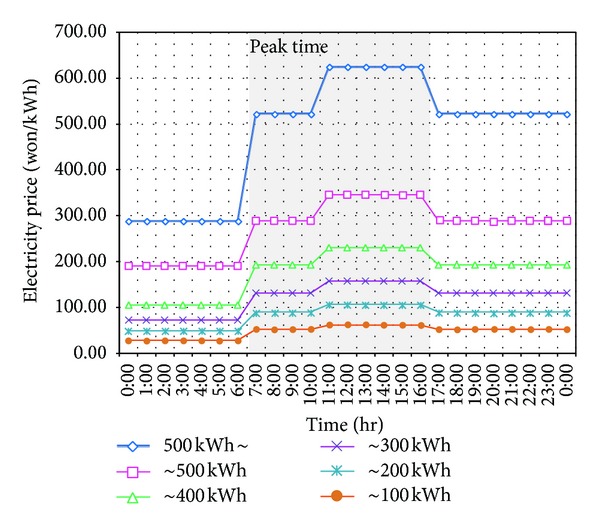
Real-time pricing system used in Jeju.

*Multilevel Peak Time*. In Figure 2, the price of electricity is classified into two regions, nonpeak and peak, but in real situations that adopt real-time pricing of electricity, multilevel price will be defined for each region. For example, Figure 3 plots the dynamic electricity pricing system adopted in Jeju Island on a trial basis. The *x*-axis represents the time slot of a day, and the *y*-axis refers to a unit price for each time slot. In this case, the price of each time region and its progressive threshold vary and the problem becomes even more complicated. Thus, an appropriate optimization technique for efficient scheduling is needed [3].

Considering these overall situations, this paper presents a new electricity usage scheduling algorithm for smart buildings that models the problem as a real-time scheduling problem and minimizes the total charge of electricity through finding a schedule based on genetic algorithms. The algorithm also guarantees the safe execution of all electricity demands in a smart building by considering their own characteristics such as the minimum execution period, operating length, deadline, and mandatory/optional execution. In order to find an optimized schedule, the proposed system uses a smart device to perform the collaboration of each device in changing the power state. That is, an application within a smart device finds a schedule that does not exceed the high price progressive threshold for each time period by the collaboration of electric devices and controls them.

## 3. A Collaborative Electricity Usage Scheduling with Genetic Algorithms

**Figure 4:**
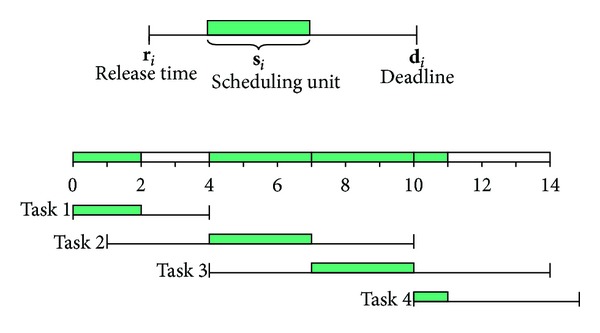
A real-time task scheduling problem.

Electricity usage scheduling of a smart building in this paper can be defined similarly as a real-time task scheduling problem which is one of the representative optimization problems in computer systems. In a real-time task scheduling problem, time-critical tasks should be serviced within certain preassigned deadlines dictated by the physical environment. As shown in Figure 4, each task has its own release time, deadline, and scheduling unit. A task can start its execution after its release time and should be serviced for the scheduling unit before its deadline. In other words, the scheduling unit of a task should be executed between the release time and the deadline. Then, the scheduling problem becomes a placement problem of each scheduling unit in its time slot.

In Figure 4, there are four tasks that have their own release time and deadline, and a valid schedule that places each task in an appropriate time slot is exemplified. In traditional real-time task scheduling problems, multiple tasks cannot be executed simultaneously as there is a single processor in the system. This is released as modern multiprocessor or multicore systems can execute multiple tasks on different cores or processors. The electricity usage scheduling problem in this paper is similar to the real-time task scheduling problem in multiprocessor systems, which can execute multiple tasks by scheduling them at the same time.

The problem of electricity usage scheduling becomes more complicated as the price of electricity is not proportional to the sum of the power usage but increases dramatically due to the progressive stage system of electricity prices. Moreover, since there are multilevel peak time regions, the base price of electricity also changes dynamically as time progresses.

As the existing real-time task scheduling problem is known to be a complicated optimization problem which is NP-hard, electricity usage scheduling is also a very complex problem to be optimized. Finding an optimal schedule in an efficient way is not known, and it can only be found by enumerating all possible schedules and then evaluating them. The complexity of the problem search domain is *O*(2^*MN*^) where *M* is the number of tasks and *N* is the number of time slots. For example, there are 23 electric devices to schedule, and if the scheduler needs to decide their on/off every 30 minutes, the number of possible schedules within a day reaches 2^23∗24∗2^, that is, 2^1104^. Even with high-end computer systems, it is almost impossible to search all these schedules to find a schedule with the minimum electricity charges.

**Figure 5:**
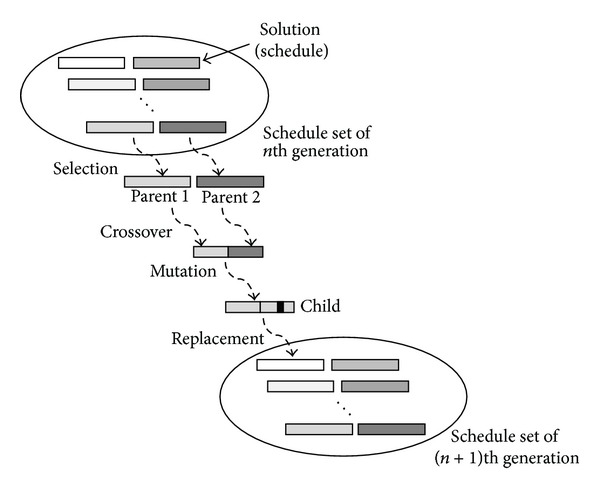
Each step of a genetic algorithm to perform electricity usage scheduling.

Thus, this paper cuts down the huge searching space using an evolutionary computation method to find an approximated schedule within a reasonable time budget. Specifically, we use a smart device to perform the genetic algorithm, which is a probabilistic search method based on natural selection and the population genetics [14].  Figure 5 depicts a brief flow of the genetic algorithm. A certain number of schedules are initially generated and they form the initial set of schedules. Among schedules in the set, two schedules are selected and then merged as one schedule by the crossover and mutation operations, and then the schedule set is evolved by replacing a schedule in the old set by the newly generated schedule. This process is repeated until the schedule set converges.

### 3.1. Encoding and Constructing Initial Scheduling Set

**Figure 6:**
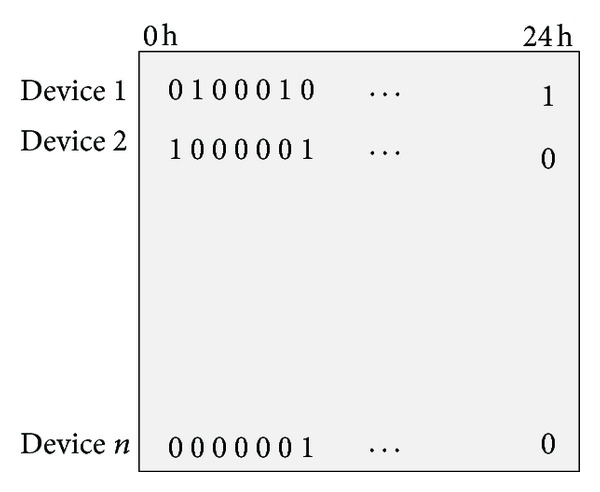
Solution encoding of the scheduling problem.

Each solution is encoded into a string in a genetic algorithm. In the scheduling problem, a solution represents one of all possible schedules that determine the state of all electric devices in a building during a day. The choice of this encoding is a major feature of a genetic algorithm. In the proposed algorithm, a solution is encoded by a matrix as shown in Figure 6. Rows and columns of the matrix represent electric devices in a building and the 24 hours of a day, respectively. The value of each entry in the matrix can be defined as 0 or 1; the value of 0 refers to the state that the device is switched off, and 1 refers to the state that it is switched on.

In some kind of electric devices, multiple power modes should be defined. For example, a rice cooker has a cooking mode that consumes much power and a warming mode that only keeps current state. The encoding scheme used in this paper accommodates these multiple power modes by separating them as different rows. That is, cooking and warming modes of a rice cooker are considered as separate devices and appropriate time constraints are set to operate them properly (refer to Figure 8). This simplifies the encoding problem compared to defining multiple values in each matrix entry.

Actually, each device has its own time scale in its power demand behavior. There are interruptible and noninterruptible scheduling cases, which differ in their degree of freedom to split the requested tasks across the available time slots. Interruptible tasks may be scheduled to several nonadjacent time slots while noninterruptible ones need consecutive time slots to complete the task. This paper considers the interrupt of a task in the scheduling by mapping an interruptible task to several uninterruptible ones with a finer-grained time slot.

The initial scheduling set is composed by generating 1000 random solutions. If a generated solution is not feasible, which refers to the situation that it does not satisfy the time constraints of each electric device, the algorithm performs adjustment operations for each row of the matrix to generate a feasible solution as follows. First, if the number of 1s in the entries is not valid, the algorithm randomly selects a column and then changes its bit. This process is repeated until the number of 1s in the entries becomes valid. Second, if a certain scheduling unit is not located between the release time and the deadline of it, the algorithm randomly moves the scheduling unit to valid locations.

### 3.2. Selection Operations

A selection operation chooses two solutions among the current generation that will be the parents of the next generation. It is based on a probabilistic rule that favors solutions performing better according to their fitness measure. In the problem domain of electricity usage scheduling, the fitness measure refers to the electricity charge of the schedule. However, this operation should avoid too much discrimination. For example, when there exist one or two extremely superior solutions, the selective pressure may focus narrowly on these extreme ones, and the characteristics from these solutions may dominate the solution space rapidly, potentially causing premature convergence to a local optimum. Thus, this paper applies the most common practice, which gives the best solution in the current generation four times more probability to be selected than the worst solution [14]. The normalized fitness function is as follows:

(1) $$\begin{array}{c} {\text{NF}}_{i}=\left(R_{w}-R_{i}\right)+\frac{\left(R_{w}-R_{b}\right)}{3}, \end{array}$$

where *R*_*w*_, *R*_*b*_, and *R*_*i*_ are the ranks of the worst solution, the best solution, and solution *i*, respectively, in the current generation. Based on the normalized fitness function, a roulette wheel selection is applied as a selection operator. It assigns each solution *i* a slot with size equal to its normalized fitness value NF_*i*_. The pointer of the wheel is a random real number ranging from 0 to ∑NF_*i*_. A solution whose slot spans the pointer is selected and becomes a parent.

### 3.3. Crossover and Mutation Operations

The idea of a crossover operation, which is generally regarded as the most important search operator in genetic algorithms, is that useful segments (i.e., partial solutions) of the selected parents should be combined to yield new solutions which will lead to better solutions over time.

**Figure 7:**
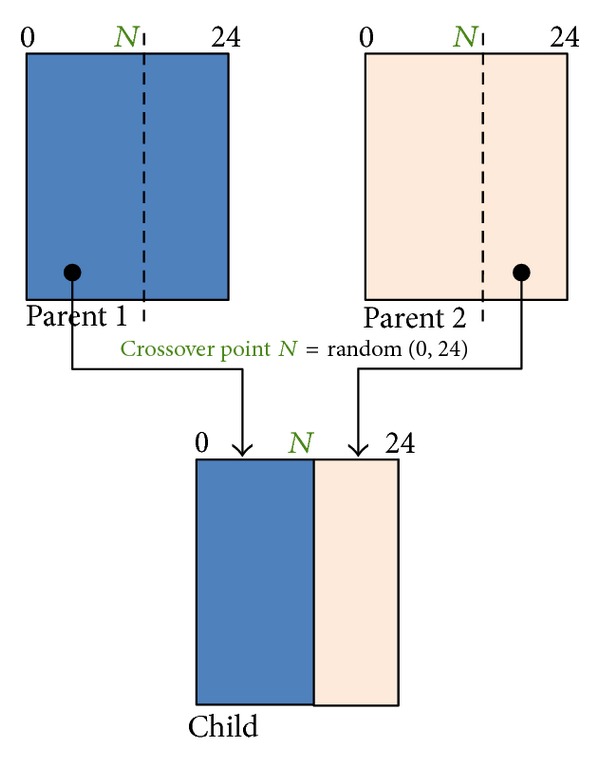
Crossover operations in the proposed scheduling algorithm.

A classical one-point crossover operation works by randomly generating a crossover point and then swapping segments of the two parent strings to produce one or two child strings. In the proposed scheme, solutions are encoded not as a string but as a matrix. Thus, one-point crossover operation is defined such that it generates a crossover point cutting the column of a matrix.  Figure 7 shows an example of the proposed crossover operation. A random number between 0 and 24 is generated and a new solution is formed by inheriting the left and right segments of parents 1 and 2, respectively.

This paper does not explicitly define mutation operations, which make a change to the generated child for the diversity, because crossover operations should include some adjustment routines that play a role of mutation. Specifically, when the classical one-point crossover is applied to the electricity usage scheduling problem, it may produce an infeasible solution; the scheduling sequence in the solution may not be feasible scheduling information such that it does not satisfy the time constraints of each device. This kind of situation happens when the crossover point cuts the sliding window of a scheduling unit. The proposed algorithm performs adjustment such that the child inherits the scheduling unit from parent 1 preferentially, and the remaining slots are filled by parent 2. If there are still some empty slots, it randomly chooses any location in the sliding window and then fills them.

### 3.4. Replacement Operations

When a child solution is generated, the new generation is produced by substituting the generated child with a solution in the current generation. This paper replaces the most inferior solution that incurs highest electricity charge in the current generation by the newly generated one, which is the most commonly used replacement operation.

## 4. Prototype Architecture and Experiments

**Figure 8:**
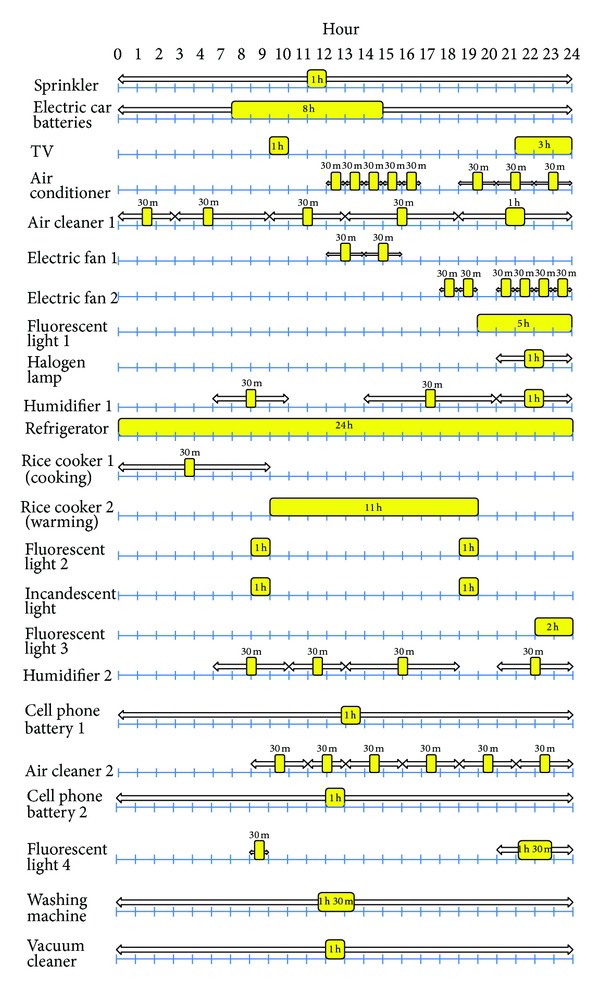
An example of electricity demands and their scheduling slots.

This section presents the performance evaluation results to assess the effectiveness of the proposed scheduling system. A set of scheduling applications running on a smart device were developed to find a possible schedule given various electricity demand conditions. The proposed scheduling system is compared with those that do not use genetic algorithms. The price of electricity used in this paper is shown in Table 1. It is benchmarked from the real-time pricing system used in Jeju and then extended to consider progressive stage systems. Figure 8 shows an example of electricity demands and constraints for each device in a smart building. In the figure, rectangular boxes represent the scheduling unit of each device to be powered-on during a day, and the horizontal arrows represent the sliding time window of the scheduling unit. For example, a rice cooker 1 requires the usage of electricity for 30 minutes between 0 and 8 a.m. As mentioned in Section 3, if a device has multiple power modes, they are encoded separately as if they are different devices.

**Figure 9:**
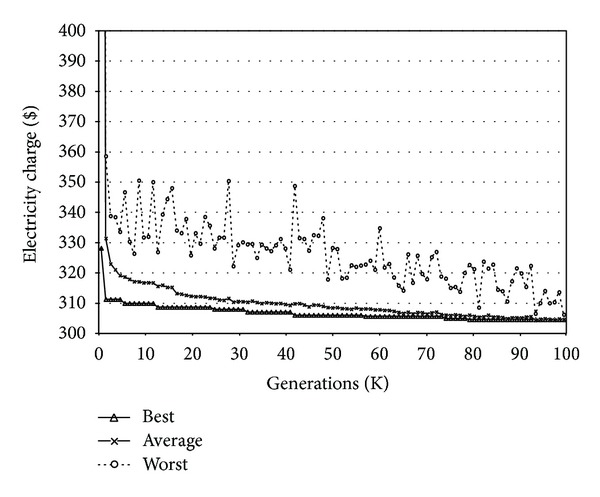
Convergence of the scheduling set as time progresses.

The experiments were performed with six different scale buildings, where the number of electricity demands is 50, 100, 150, 200, 250, and 300, respectively. Before showing the comparison results, Figure 9 plots the fitness measure of the best and the worst solutions among 1000 solutions in the current searching space and their average as the generation progresses. As shown in the figure, the quality of solutions improves significantly according to the evolution of the generation, and finally they converge.

**Figure 10:**
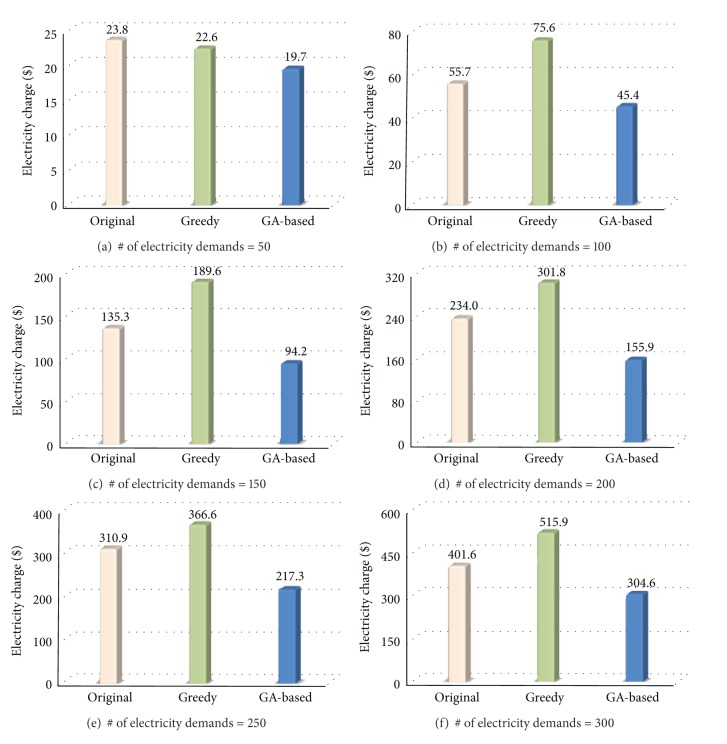
Comparison of electricity charges.

Figure 10 shows the electricity charges of the proposed GA-based scheduling system and the original system that does not use it. In the original system, each scheduling unit is randomly located among the release time and the deadline. For comparison purpose, the Greedy scheduler is also simulated. The Greedy scheduler locates each scheduling unit to the time slot of which the electricity price is lowest.

As shown in the figure, the proposed scheduling system performs the best irrespective of the building scale. The performance gain of GA-based scheduling is 27.0% on average and up to 36.4% compared to the original scheduling system. In particular, the proposed scheduling system exhibits excellent performance in a large scale building with the number of electricity demands being more than 150. The reason is that GA-based scheduling places most electricity demands to nonpeak time zones but it also tries not to exceed the high price progressive threshold. When the number of electricity demands increases, it is more likely that the sum of electricity usage exceeds the progressive threshold. The proposed system alleviates this situation by collaborating the devices to be scheduled.

The Greedy scheduler performs slightly better than the original scheduling system when the number of electricity demands is 50. However, the performance of the Greedy scheduler becomes even worse than that of the original scheduler as the building scale becomes large. Specifically, the performance degradation of the Greedy scheduler is 24.3% on average and up to 40.1% compared to the original scheduling. This may seem to be a strange result, but it could happen when considering the principle of progressive stage systems. Though the Greedy scheduler avoids the peak time efficiently, it may exceed the high price progressive threshold of other time slots as it does not consider the load balancing for those slots.

**Figure 11:**
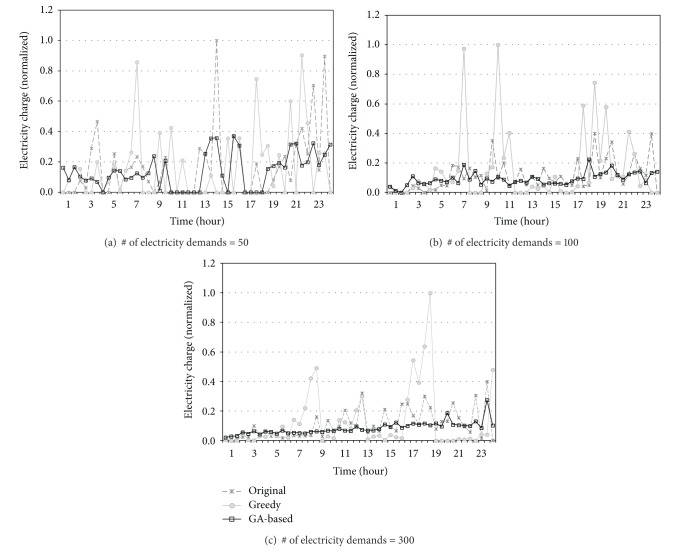
Electricity charges of each time slot of a day.

For the exact analysis of this interesting result, the electricity charge of each time slot of a day is also examined.  Figure 11 plots the normalized electricity charge of the three schedulers as time progresses within a day. As shown in the figure, the proposed GA-based scheduling system exhibits relatively uniform electricity charges irrespective of the time zone. The reason behind such balancing is that it finds a schedule that does not exceed the threshold of progressive stage system at all time slots including nonpeak zones as well through collaborative scheduling. Even though the Greedy scheduler avoids most of peak time, certain time zones in which the charge of Greedy sharply increases can be observed.

**Figure 12:**
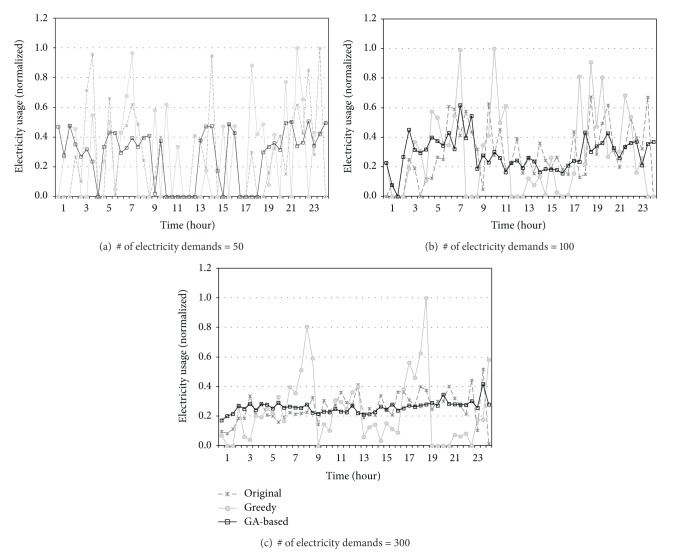
Electricity usage of each time slot of a day.

Figure 12 shows the change of the electricity usage for the three scheduling systems as time progresses. As shown in the figure, the shapes of the graphs are mostly similar to those of Figure 11. It can be observed that the proposed scheduler exhibits relatively balanced usage of power, which is consistent with the electricity charge results shown in Figure 11. One interesting feature that appeared in this figure is that the GA-based scheduling algorithm produces different shapes of electricity usage depending on the number of electricity demands. When the number of devices is 50, the GA-based algorithm minimizes the electricity usage at the peak time, while when the number of devices becomes 300, it exhibits more uniform distribution of power usage. This implies that when the building scale is small, avoiding peak time is effective in reducing the total electricity charge, while controlling the electricity usage of each time slot not to exceed the progressive threshold is important when there are many electricity demands to be scheduled. It can be observed again from Figure 12 that the electricity usage of the Greedy scheduler is certainly reduced at peak hours of 11 a.m. to 5 p.m. As aforementioned, however, this effort does not result in the reduction of the electricity charges eventually.

## 5. Related Works

With the evolution of power systems to smart grid, there have been plenty of studies on intelligently managing the electricity at the demand side for energy savings. Mohsenian-Rad et al. present an autonomous demand-side energy management system when the utility company can adopt adequate pricing tariffs that differentiate the energy usage in time and level. They formulate an energy consumption scheduling a game and find the optimal strategy that can minimize the energy costs by reducing the peak-to-average ratio of the total energy demand [15].

Li et al. propose a distributed algorithm for the utility company and the customers to jointly compute optimal prices and demand schedules [16]. They model the energy demand fluctuating of household appliances as a cost-and-benefit function and dynamically control the appliances to reduce peak load and variation in demand. Gatsis and Giannikis present a formulation for scheduling demand response among residences when the adjustable power consumption is allowed with the energy charging system [17]. As the adjustable power consumption does not increase a total energy requirement but provides satisfaction to the end-user, the utility company and the end-users exchange pricing signals and find the optimal schedule to adjust the power consumption across the scheduling horizon.

As the demand side energy management receives increasing attention by research and industry, Palensky and Dietrich analyze and summarize various types of DSM, giving an outlook on the latest demonstration projects in this domain [13]. Abras et al. suggest MAHAS (Multiagent Home Automation System) that adjusts power consumption according to inhabitant comfort and cost criteria [18]. They make use of a multiagent approach that divides the power management problem into subproblems and each agent tries to solve its own problem independently to finally find a global solution for the whole problem.

Derin and Ferrante discuss a scheduling problem for household tasks to reduce the cost for energy consumption when users have options to select preferred electricity supplier companies and are also given the availability of locally generated power [19]. They build a system model formulating source of power, dynamic pricing, and tasks with deadlines and define a scheduling problem based on it. They present that finding the optimal schedule through exhaustive search is not feasible and thus need an efficient heuristic that would work for large number of tasks and time slots.

Zhang et al. propose an optimal scheduling algorithm that reduces the energy cost and peak demand in multiple smart home environments [20]. Assuming different electricity tariffs, actuation time of appliances, and forecasted renewable energy output, they judiciously control the operation and electricity consumption tasks to minimize a one-day expected energy consumption cost based on the mixed-integer linear programming. Lee et al. propose a power consumption scheduler for smart grid homes to reduce the peak load in individual homes and the system-wide power transmission networks [21]. Similar to studies in this paper, they also model home appliances as real-time tasks defined by actuation time, operation length, and deadline. However, their work is different from the studies performed in this paper in that they find an optimal solution by traversing all the feasible allocations for a task set, while this paper relies on a heuristic search mechanism based on genetic algorithms, which finds a nearly optimal solution in a short time.

## 6. Conclusions

This paper presented a new electricity usage scheduling algorithm for smart buildings that use real-time pricing of electricity in the emerging smart grid environments. The proposed algorithm uses a smart device to perform a scheduling application. The scheduling application makes use of genetic algorithms to schedule electric devices in the building according to the change of electricity prices. Experimental results with various electricity demand conditions show that the proposed algorithm reduces the electricity charges of a smart building by 27.0% on average and up to 36.4%. It is expected that the proposed scheduling will contribute to alleviating the disparity of power usage by reducing the electricity usage at peak time. It will be also helpful to the success of dynamic pricing, which highly depends on the consumer's actual response to the time-varying prices. Adopting smart devices to perform the electricity usage scheduling will expand the application domain of smart devices further.

## Figures and Tables

**Table 1:** Electricity Prices used in the experiments (won/kWh).

	~100 kWh	~200 kWh	~300 kWh	~400 kWh	~500 kWh	500 kWh~
00:00~07:00	28.95	49.35	73.22	106.37	191.15	288.29
07:00~11:00	52.40	89.30	132.50	192.50	288.90	521.70
11:00~17:00	62.74	106.93	158.65	230.49	345.92	624.67
17:00~24:00	52.40	89.30	132.50	192.50	288.90	521.70
